# Population characteristics of golden retriever lifetime study enrollees

**DOI:** 10.1186/s40575-017-0053-5

**Published:** 2017-11-15

**Authors:** Melissa Simpson, Erin Searfoss, Sharon Albright, Diane E. Brown, Barbara Wolfe, Nancy K. Clark, Susan E. McCann, David Haworth, Mike Guy, Rod Page

**Affiliations:** 1 Morris Animal Foundation, 720 South Colorado Boulevard, Suite 174-A, Denver, CO 80243 USA; 2 American Kennel Club Canine Health Foundation, P.O. Box 900061, Raleigh, NC 27675 USA; 3 Roswell Park Cancer Institute, Elm and Carlton Streets, Buffalo, NY 14263 USA; 4 PetSmart Charities, 19601 North 27th Avenue, Phoenix, AZ 85027 USA; 5 Jaguar Pharmaceuticals, 201 Mission Street, Suite 2375, San Francisco, CA 94105 USA; 6 Colorado State University, College of Veterinary Medicine and Biomedical Sciences, Fort Collins, CO 80523-1620 USA

## Abstract

**Background:**

Studying cancer and other diseases poses a problem due to their protracted and multifactorial nature. Prospective studies are useful to investigate chronic disease processes since collection of lifestyle information, exposure data and co-incident health issues are collected before the condition manifests. The Golden Retriever Lifetime Study is one of the first prospective studies following privately-owned dogs throughout life to investigate the incidence and risk factors for disease outcomes, especially cancer.

Owners of golden retrievers in the contiguous United States volunteered their dogs in early life. Owners and veterinarians complete online questionnaires about health status and lifestyle; dogs undergo a physical examination and collection of biological samples annually. The data presented summarize the initial study visits and the corresponding questionnaires for 3044 dogs in the cohort.

**Results:**

The median age of dogs at enrollment was 14.0 months (interquartile range (IQR): 8–20 months). Approximately half of the population had undergone gonadectomy by their initial study visit. Medical conditions reported at enrollment consisted primarily of integumentary, gastrointestinal and urinary dysfunction. A large majority of the dogs have a record of having received preventive care (vaccines, parasiticides, flea and heartworm prevention) by the time of the initial study visit. Clinical pathology data were unremarkable.

**Conclusions:**

This study represents one of the first lifetime observational investigations in veterinary medicine. The population characteristics reported here indicate a healthy cohort of golden retrievers cared for by owners committed to their dogs’ health. Data acquired over the study period will provide valuable information about genetic, dietary and environmental risk factors associated with disease in golden retrievers and a framework for future prospective studies in veterinary medicine.

**Electronic supplementary material:**

The online version of this article (10.1186/s40575-017-0053-5) contains supplementary material, which is available to authorized users.

## Plain English summary

Identifying risk factors for cancer is challenging due to the complexity of the disease process and how long it takes for clinical signs associated with disease to become apparent. Longitudinal studies (studies that follow subjects over time with the intention of gaining insight into the development of disease outcomes, ideally study subjects just “live their lives” and are asked to periodically check in to report their experiences) are useful to investigate chronic disease since exposures are collected before the condition manifests. The Golden Retriever Lifetime Study is one of the first longitudinal studies following dogs to investigate the incidence of and risk factors for cancer. Owners of 3044 golden retrievers in the United States volunteered their dogs for this observational cohort. Owners and veterinarians complete online questionnaires about health status and lifestyle of dogs who also undergo a physical examination and collections of biological samples annually. The data presented in this paper are from the dogs’ initial study visit. The median age of dogs at enrollment was 14 months; approximately half had undergone spay or neuter surgery. Medical conditions reported at enrollment consisted of skin, digestive and urinary dysfunction and were all minor. A majority of the dogs have a record of receiving preventive care (vaccines, parasiticides, flea, and heartworm prevention). Blood work, urinalysis, and fecal parasite test results were largely unremarkable. This study represents one of the first lifetime observational investigations in veterinary medicine. The population characteristics reported here indicate a healthy cohort of golden retrievers cared for by committed owners. This study will provide valuable information about risk factors associated with cancer in golden retrievers and a framework for future prospective studies in veterinary medicine.

## Background

Cancer and other diseases with chronic onset are the result of a complex interplay between environmental and intrinsic host factors. The onset of such diseases may be insidious and are often accompanied by a protracted subclinical prodrome [1]. These attributes make identifying potential etiologies challenging. Identifying putative exposures and characterizing the importance of sensitive period effects, length of exposure, dose, and interactions between exposures is especially challenging with complex and multifactorial diseases. Prospective studies address some of these issues as exposure data are collected in real time and prior clinical manifestations of disease. This feature mitigates the potential for bias that accompanies other study designs and ensures that exposure preceded clinical disease and that both the timing and extent of exposure are collected proximally to when they occurred.

Because of domestic dogs’ abbreviated life-span relative to humans, it is more feasible to collect exposure and outcome data over the entire life-course. This attribute makes investigating phenomena such as sensitive period effects and cumulative exposures efficient relative to human studies that are similarly structured. While prospective studies are well-known and regularly employed to study disease etiology and potential risk factors in people [2], to date, large-scale life course veterinary studies have been scarcely reported. Four prospective studies in dogs are currently underway: The Golden Retriever Lifetime Study (GRLS), the 9/11 Medical Surveillance Study of search dogs deployed to the terrorist attack sites [3, 4], *Generation Pup*, a study following dogs of all breeds from puppy- to adulthood, and *Dogslife*, a web-based study, which reported data regarding about 4300 Labrador Retrievers in the UK [5–7] and has currently enrolled more than 7000 dogs. The data presented herein are from the GRLS.

The ongoing impetus for the GRLS is to estimate the incidence of and genetic, nutritional, and environmental risk factors for four fatal cancers in golden retrievers: high-grade mast cell tumors, osteosarcoma, hemangiosarcoma, and lymphoma [8–10]. In addition to collecting data about these outcomes, study participants and their veterinarians are asked to report annually about many aspects of the dogs’ lives including nutrition, indoor and outdoor environmental exposures, reproductive status and history, preventive health care, behavior and temperament, exercise habits, results of an annual physical examination, clinical pathology laboratory results, and all major health events. All clinical pathology data are collected by a single reference laboratory. All clinical pathology results were shared with owners through their personal veterinarians and, when needed (Eg: positive heartworm results), appropriate treatments were pursued as per the normal veterinary/client/patient relationship.

The purpose of this report is to present descriptive characteristics of a cohort of 3044 golden retrievers at enrollment.

## Methods

### Study population

The data presented in this study were collected on 3044 golden retrievers enrolled in GRLS. Details about the structure of the study, recruitment and enrollment process and data collection have been previously published [11]. Briefly, we recruited 3044 privately-owned dogs living in the contiguous United States that were between six months and two years of age at the time of entry into the study. Knowledge of prior pedigree for at least two generations was required. Both the owner and veterinarian gave informed consent to participate and committed to the requirements of the study which include annual internet-based questionnaires (including both structured and free text response options) for both the owner and veterinarian, complete veterinary physical examinations, and biological sample collection (whole blood and DNA, serum, urine, feces, hair, and toenails).

In addition, at the time of a diagnosis of any malignancy (not solely the four primary outcomes), another set of biological samples are obtained in addition to a physical exam report, diagnostic laboratory reports, and histopathology samples, when appropriate.

Recruitment was started in 2012 and all requirements outlined above were completed on 3044 (100%) dogs in March 2015; our target was 3000 dogs and enrollment closed when that goal was met. The additional 44 dogs were enrolled because they completed enrollment requirements before enrollment officially closed. Body condition score was reported by veterinarians using the Purina® nine point scale [12].

The study protocol and participation requirements were reviewed and approved by Morris Animal Foundation’s appointed Animal Welfare Advisory Board and informed consent was obtained from all owners and veterinarians prior to enrollment.

### Analysis

The data presented are from the owner questionnaire, the initial, qualifying clinical visit captured in the veterinarian questionnaire, and clinical pathology results obtained at the time of enrollment. Because the data are descriptive, no formal statistical tests were performed. To ascertain veterinarian-reported medical conditions, we asked the question by body system, “Indicate if the dog has been diagnosed with a given condition in the past 12 months.” To account for interval censoring in the reporting of medical conditions when calculating incidence density, we estimated that all diagnoses occurred 6 months prior to the initial clinical visit for dogs 6 months or older at the time of the visit. For dogs younger than 6 months at the time of their visit, we used the assumption that all diagnoses occurred at age (in months)/2. All tables and figures were generated using commercially available statistical software.^1^

## Results

**Fig. 1:**
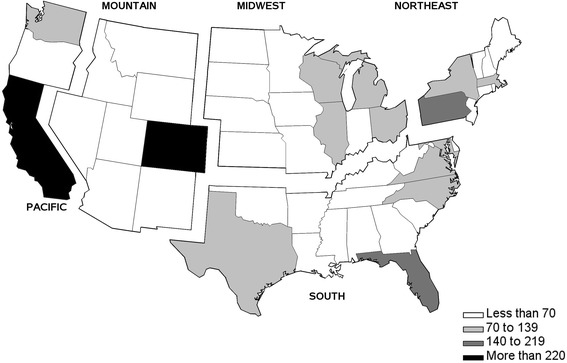
Recruitment regions and number of dogs enrolled by state in the Golden Retriever Lifetime Study

We defined five regions of the contiguous U. S. to direct recruitment efforts: Northeast, South, Midwest, Mountain, and Pacific (Fig. 1). In addition, Fig. 1 indicates the general quantitative range of dogs enrolled by state. California had the largest number enrolled (*n* = 278) while Colorado had the largest enrolled per human capita (*n* = 255; 5 dogs/100,000 people) and most states have fewer than 70 dogs enrolled.

Table 1 contains basic descriptive information about the study dogs at the initial clinical visit. The median age of study dogs at the initial clinical visit was 14.0 months (IQR 8.3–20.3). Overall, 578 (19%) owners reported having pet health insurance. Ninety five percent of the dogs in this study were acquired from breeders. All but 3% of study dogs sleep in the owner’s house and 84% of owners report that the primary job of their dog is to be a pet or companion rather than primarily a working or service dog. Of the 1137 (37%) dogs who had undergone gonadectomy prior to their first study visit, the majority (97%) were elective rather than the result of a medical or behavioral indication. Among male dogs, cryptorchid testicles were the most common non-elective indication for gonadectomy (85 dogs, 16% of neutered males). Four female dogs underwent gonadectomy for medical indications, including three dogs with cystitis and one with metritis.

**Table 1:** Descriptive characteristics of 3044 Golden Retriever Dogs participating in the Golden Retriever Lifetime Study at their initial clinical study visit

Characteristic	Region				
	Northeast	Midwest	South	Mountain	Pacific
	*n* = 635	*n* = 722	*n* = 841	*n* = 413	*n* = 433
Sex					
*n*(%) spayed female	118 (19)	151 (21)	182 (22)	86 (21)	101 (23)
*n*(%) intact female	203 (32)	201 (28)	231 (27)	109 (26)	122 (28)
*n*(%) neutered male	88 (14)	127 (18)	132 (16)	87 (21)	82 (19)
*n*(%) intact male	226 (36)	243 (34)	296 (35)	131 (32)	128 (30)
Median age at spay/neuter in months (± IQR)	6.8 (6.1–10.3)	6.4(5.5–8.4)	6.7(5.6–9.4)	6.6(5.6–9.1)	7.1(5.9–10.8)
*n*(%) reporting elective spay/neuter	199 (97)	273 (98)	309 (98)	165 (95)	174 (95)
*n*(%) Registered with the American Kennel Club	547 (86)	603 (84)	741 (88)	321 (78)	367 (85)
*n*(%) with pet health insurance	150 (24)	79 (11)	148 (18)	91 (22)	110 (25)
*n*(%) acquired from a breeder (includes bred by current owner)	612 (96)	688 (95)	791 (94)	374 (91)	418 (97)
*n*(%) sleeping in the house	629 (99)	697 (97)	826 (98)	403 (98)	411 (95)
*n*(%) primary activity is companion or pet	520 (83)	573 (80)	697 (84)	360 (88)	371 (87)

Owner-reported environmental and lifestyle exposures are shown in Table 2. Most dogs (83%) receive both ectoparasite (flea and tick) and heartworm prevention, 15% only receive heartworm prevention, 5% only receive ectoparasite prevention, and 6% do not receive either medication. Sixty four percent of Americans as a whole report purchasing flea prevention products [13]. Of the dogs who receive preventive medications, 1805 (59%) receive ectoparasite preventive topically and 2454 (81%) receive heartworm prevention orally. Almost 100% of dog owners report giving their dogs treated (Eg: municipal water) or filtered well water as their source of drinking water (these water sources are considered safe for human consumption) and that they live in a suburban environment (60%); urban living conditions are the least frequently reported (10%). A small portion of participating dogs (6%) are exposed to secondhand smoke and exposure time ranges from 1 h per day to 24 h per day. Most owners feed a commercial diet (85%); among owners who report feeding a home-prepared diet, 257 (55% of home-prepared feeders, 8% of the entire cohort) report feeding a diet that is at least partially raw.

**Table 2:** Owner-reported environmental and lifestyle exposures among 3044 Golden Retriever Dogs at the time of first owner questionnaire

Characteristic	*n*	%
Flea prevention	2389	79
Seasonal prevention	598	20
Year-round prevention	1791	59
Heartworm prevention	2702	89
Seasonal prevention	611	20
Year-round prevention	2091	69
Drinking water source		
Municipal	809	27
Well, treated or filtered	2211	73
Well, untreated	24	1
Swims at least weekly	1200	39
Drink or eat from a plastic bowl	683	22
Eat feces	777	26
Owner reported physical activity level		
Little or none	28	1
Moderate	1469	48
Very active	1547	51
Owner reported insecticide or herbicide treatment		
In the house	662	22
In the yard	1136	37
Owner reported grass eating behavior		
Frequent	823	27
Infrequent	1701	56
Never	519	17
Type of residence		
Urban	317	10
Suburban	1837	60
Rural	890	29
Second hand smoke exposure	185	6
Median hours exposed per day (±IQR)	3.5	4.1
Type of dog food		
Commercially prepared	2580	85
Home prepared	392	13
Both commercially and home prepared	72	2

The results of veterinary physical examination are shown in Table 3. The median body condition score (BCS) was 5 (IQR 5–6), 868 (29%) dogs had a BCS of 5.5/9 (i.e. overweight) or higher. The median height at the withers for all sexes combined was 57.2 cm (IQR: 54.6–61.0 cm); median weight for all sexes combined was 27.8 kg (IQR: 24.4–31.6 kg). Overall, 1822 (59%) of dogs had a normal physical exam. The most common abnormalities noted on physical exam at the initial clinical visit were dental tartar (*n* = 336, 11%), otitis externa (*n* = 309, 10%), and skin conditions (*n* = 259, 9%).

**Table 3:** Results from physical examination on 3044 Golden Retriever dogs at their initial clinical study visit

Characteristic	Sex			
	Male intact	Male neutered	Female intact	Female spayed
	*n* = 1024	*n* = 516	*n* = 866	*n* = 638
Median Purina® Body Condition Score (±IQR)	5 (5–5)	5(5–6)	5(5–5)	5(5–6)
Median weight in Kg (±IQR)	29.1 (25.5–32.3)	32.5 (29.1–35.9)	24.8(21.7–27.4)	27.5(24.8–30.8)
Median height at withers in cm (±IQR)	58.4 (55.9–61.0)	61.0 (58.4–63.5)	54.6 (53.0–57.2)	57.2 (54.6–60.1)
Median age in months at exam (±IQR)	11.0 (7.3–17.1)	18.5(13.7–23.3)	11.1 (7.4–17.6)	16.9 (12.2–22.5)
n(%) with a normal physical exam	633 (62)	268 (52)	556 (64)	365 (57)

Table 4 shows prevalence and incidence density of diagnoses reported in the 12 months preceding the initial clinical visit by body system and the most commonly reported diagnosis with each system. Study dogs accrued 15,492.5 dog months (mean of 5 months per dog) at risk when calculating incidence density of veterinary-reported conditions. Problems of the integumentary system were the most commonly reported. Otitis externa was the most common diagnosis, followed by enteritis.

**Table 4:** Prevalence and incidence density of diagnoses by body system and the most common diagnosis within systems in a cohort of 3044 Golden Retriever dogs during the 12 months preceding their initial clinical study visit

Body system	Prevalence (diagnoses/3044 dogs enrolled)	Incidence density
		(new diagnoses per 1000 dog-months)
Integumentary (*n* = 943)	0.31	61.51
Otitis externa (*n* = 561)	0.18	36.21
Gastrointestinal (*n* = 578)	0.19	36.79
Enteritis (acute and/or self-limiting) (*n* = 365)	0.12	23.37
Urinary (*n* = 274)	0.09	17.94
Bladder infection/Cystitis (*n* = 273)	0.09	17.69
Musculoskeletal (*n* = 213)	0.07	14.72
Pain or lameness (*n* = 59)	0.02	6.45
Ophthalmic (*n* = 161)	0.05	10.39
Conjunctivitis (*n* = 121)	0.04	7.75
Reproductive (*n* = 91)	0.03	6.52
Cryptorchid (unilateral or bilateral)(*n* = 61)	0.02	4.84
Neurologic (*n* = 30)	0.01	1.10
Epilepsy (*n* = 7)	0.00	0.45

Eighty four percent (*n* = 2549) of dogs received at least one dose of American Animal Hospital Association recommended core vaccines (rabies, parvovirus, distemper, and adenovirus) [14]; 90% of dogs received at least one vaccination against rabies virus, and 72 (2%) dogs received both a one-year and a three-year rabies vaccine during the preceding 12 months. The most common non-core vaccines reported were four-way leptospirosis (47%, *n* = 1444), and intranasal Bordetella (36%, *n* = 1106). Eighteen percent (*n* = 549) of dogs received a vaccine against *Borrelia burgdorferi*. Two hundred ninety nine (10%) dogs did not have a record of having received a vaccination on or prior to the initial clinical study visit.

As part of the annual study visit, a serum chemistry panel, total T4, heartworm antigen test, complete blood count (CBC), urinalysis, and fecal pathogens analysis were performed. Forty percent (*n* = 1198) of dogs had all values included on a CBC within the normal limits established by the reference laboratory. On serum chemistry, 1828 (60%) of dogs had all values within reference ranges. Quantitative urinalysis results were 100% within reference laboratory normal limits; eleven dogs (<1%) tested positive for heartworm antigen; 64 (2%) dogs had low total T4 (minimum value: 0.49 μg/dl; reference range: 0.8–3.5 μg/dl), and 282 (9%) dogs had at least one parasite on fecal flotation. A list of the clinical pathology tests, normal ranges, and units can be found in Additional file 1: Table S1.

## Discussion

This cohort of golden retrievers will be followed through their life-course to estimate the incidence of cancer and other major diseases or disorders and investigate the risk factors for these conditions. As the study progresses, this population of dogs will receive care from their primary and referral veterinarians who will systematically contribute data to the study. This attribute will allow for collection of data from a population of dogs whose owners pursue a wide spectrum of veterinary care.

A variety of owner-reported exposures and lifestyles will allow investigation of the association between these exposures and important health outcomes. While there is potential for misclassification of exposure with owner-reported data [15], annual collection of the same information over time will allow us to study trends. Future nested studies using the data and samples collected on this cohort will examine a multitude of important associations, including nutrition. In addition, as novel biomarkers of health and disease are developed, we will utilize prospectively collected samples to study these biomarkers as a way to potentially identify disease earlier and give insight into etiology of disease.

At the time of the initial clinical study visit, study dogs were largely healthy with skin and gastrointestinal problems being the most commonly reported veterinary diagnoses. These conditions are commonly diagnosed in young dogs [16, 17]. Clinically significant abnormalities on routine laboratory tests were rare, and all dogs were free from chronic or life-limiting disease at the time of initial physical exam, as this was an exclusion criterion. A common abnormality found was low total T4 on the serum chemistry panel. As the study progresses, these abnormalities and all major illnesses reported by veterinarians will be adjudicated by a panel of subject matter experts in order to ensure standardized disease definitions.

About half of the dogs were gonadectomized at the initial clinical study visit, compared to the national average of 85% [13]. The importance of reproductive hormones in the health and disease of these dogs will be investigated as the study progresses. The median height and weight parameters for participating dogs were within breed standard regardless of gender or reproductive status (Males: 58–61 cm at the withers and 29.5–34 kg, Females: 55–57 cm at the withers and 25 to 29.5 kg) [18]. As the study progresses, it will be important to compare mature height and weight between intact and gonadectomized dogs as well as the effect of age at gonadectomy on these parameters. In addition to height and weight, reproductive hormone exposure [number of estrous cycles (females) or years (males) prior to gonadectomy] will be studied in the context of health and disease outcomes. This will have important practical implications for practicing veterinarians when counseling their clients about if or when to get their dogs reproductively altered.

This study was designed to operate until 500 combined cases of the four primary outcomes (high grade mast cell tumor, hemangiosarcoma, osteosarcoma, and lymphoma) occur. We estimated that this number of cases will take about ten to twelve years to develop. During the intervening years we will continue to collect owner- and veterinarian-reported data and biological samples; the pairing of clinical data and samples will facilitate many novel etiologic investigations into many important clinical outcomes. While we designed the study to have sufficient power to study the four primary cancer outcomes, we will collect information about many disease outcomes.

While this study has the advantage that dogs were recruited from a general population with enrollment stratified by region and gender, we still have a highly selected population of dogs and the applicability of study results to other canine populations remains to be determined. Owners are encouraged to pursue diagnostic and treatment for all conditions after careful consultation with their primary veterinarian so we do not expect that participation in the study will influence diagnosis or course of clinical disease but the possibility of participant bias is remains. A similar study in Labrador retriever dogs may provide a means to compare results and assess the degree to which these data can be applied to other populations [6].

Retaining study dogs and maintaining owner engagement will be a crucial element in ensuring that data are representative of the original cohort. Ongoing retention efforts include encouraging participant engagement through social media, special events to recognize participants and dogs, and technical support. At the time of submission of this manuscript, 28 (<1%) dogs had been withdrawn from the study, 51 had died of various conditions including cancer, and 84% of the cohort is fully compliant with study requirements. This compliance rate is high compared to cohort studies involving a similar scope and time frame in humans [19, 20]. Factors reported at enrollment that were associated with one-year compliance in our cohort included sleeping in the owners’ bedroom, regular grooming, and current with core vaccinations [21].

## Conclusion

The value of long-term prospective studies in the advancement of human and veterinary health is difficult to understate; many important disease associations have arisen from those studies and they continue to inform treatment and prevention recommendations and public health policy [22–24]. The need for similar data in companion animals is equally important and this study is designed to address some of the gaps in evidence-based recommendations for veterinary medicine.

## Additional file

Additional file 1: Table S1. List of clinical pathology tests routinely performed, the reference ranges and units. (DOCX 14 kb)

## Footnotes

1 SAS version 9.4 (SAS Institute Inc., Cary, NC, USA)
