# Supplementary material for: Population characteristics of golden retriever lifetime study enrollees
Source: Canine Genet Epidemiol. 2017 Nov 15;4:14. doi: 10.1186/s40575-017-0053-5 (PMC5688750; doi:10.1186/s40575-017-0053-5)
Supplement: Additional file 1: Table S1. — List of clinical pathology tests routinely performed, the reference ranges and units. (DOCX 14 kb) [file 40575_2017_53_MOESM1_ESM.docx]

Supplemental table. List of clinical pathology tests routinely performed, the reference ranges and units.

| TEST | Reference Range | Units |
| --- | --- | --- |
| Complete Blood Count | | |
| WBC | 4.0-15.5 | 10^3^/μl |
| RBC | 4.8-9.3 | 10^3^/μl |
| Hemoglobin | 21.1-20.3 | g/dl |
| Hematocrit | 36-60 | % |
| MCV | 58-79 | fL |
| MCH | 19-28 | pg |
| MCHC | 30-38 | g/dL |
| Platelet Count | 170-400 | 10^3^/μl |
| Platelet Estimate | Adequate |  |
| Neutrophils | 60-77 | % |
| Bands | 0-3 | % |
| Lymphocytes | 12-30 | % |
| Monocytes | 3-10 | % |
| Eosinophils | 2-10 | % |
| Basophils | 0-1 | % |
| Absolute Neutrophils | 2060-10600 | /ul |
| Absolute Bands | 0-300 | /ul |
| Absolute Lymphocytes | 690-4500 | /ul |
| Absolute Monocytes | 0-840 | /ul |
| Absolute Eosinophils | 0-1200 | /ul |
| Absolute Basophils | 0-150 | /ul |
| Heartworm Antigen | Negative |  |
| Ova and Parasites With Centrifugation | Negative |  |
| Superchem | | |
| Total Protein | 5.0-7.4 | g/dL |
| Albumin | 2.7-4.4 | g/dL |
| Globulin | 1.6-3.6 | g/dL |
| A/G Ratio | 0.8-2.0 | Ratio |
| AST (SGOT) | 15-66 | U/L |
| ALT (SGPT) | 12-118 | U/L |
| Alk Phosphatase | 5-131 | U/L |
| GGTP | 1-12 | U/L |
| Total Bilirubin | 0.1-0.3 | mg/dL |
| Urea Nitrogen | 6-31 | mg/dL |
| Creatinine | 0.5-1.6 | mg/dL |
| BUN/Creatinine Ratio | 4-27 | Ratio |
| Phosphorus | 2.5-6.0 | mg/dL |
| Glucose | 70-138 | mg/dL |
| Calcium | 809-11.4 | mg/dL |
| Magnesium | 1.5-2.5 | mEq/L |
| Sodium | 139-154 | mEq/L |
| Potassium | 3.6-5.5 | mEq/L |
| Na/K Ratio |  |  |
| Chloride | 102-120 | mEq/L |
| Cholesterol | 92-324 | mg/dL |
| Triglycerides | 29-291 | mg/dL |
| Amylase | 290-1125 | U/L |
| Lipase | 77-695 | U/L |
| CPK | 59-895 | U/L |
| Total T4 | 0.8-3.5 | ug/dL |
| Urinalysis | | |
| Color |  |  |
| Appearance | Clear |  |
| Specific Gravity | 1.015-1.050 |  |
| pH | 5.5-7.0 |  |
| Protein | Negative |  |
| Glucose | Negative |  |
| Ketone | Negative |  |
| Bilirubin | Negative to 1+ |  |
| Blood | Negative |  |
| WBC | 0-3 | / High powered Field |
| RBC | 0-3 | / High powered Field |
| Casts |  | /Low powered FIeld |
| Crystal |  | / High powered Field |
| Bacteria | None | / High powered Field |
| Epithelial |  | / High powered Field |
